# Saliva-Based Biosensors: Noninvasive Monitoring Tool for Clinical Diagnostics

**DOI:** 10.1155/2014/962903

**Published:** 2014-09-08

**Authors:** Radha S. P. Malon, Sahba Sadir, Malarvili Balakrishnan, Emma P. Córcoles

**Affiliations:** ^1^Faculty of Biosciences and Medical Engineering (FBME), Universiti Teknologi Malaysia, Building VO1, Block A, Level 5, Room 27, 81310 Skudai, Johor, Malaysia; ^2^Faculty of Mechanical Engineering (FKM), Universiti Teknologi Malaysia, 81310 Skudai, Johor, Malaysia

## Abstract

Saliva is increasingly recognised as an attractive diagnostic fluid. The presence of various disease signalling salivary biomarkers that accurately reflect normal and disease states in humans and the sampling benefits compared to blood sampling are some of the reasons for this recognition. This explains the burgeoning research field in assay developments and technological advancements for the detection of various salivary biomarkers to improve clinical diagnosis, management, and treatment. This paper reviews the significance of salivary biomarkers for clinical diagnosis and therapeutic applications, with focus on the technologies and biosensing platforms that have been reported for screening these biomarkers.

## 1. Introduction

Common clinical diagnosis is based on the determination of blood biomarkers. However, this is an invasive procedure, too aggressive for certain patients. Saliva sampling is relatively simple and the presence of various disease-signalling biomarkers in saliva has meant that it can accurately reflect normal and disease states in humans. Although saliva collection and determination present some disadvantages, it has been recognised as an attractive diagnostic fluid with an increasing amount of assay developments and technological advancements for the detection of various salivary biomarkers.

In humans, oral fluid originates mainly from three pairs of major salivary glands (parotid, sublingual, and submandibular) and a large number of minor salivary glands. It also contains fluids from nonglandular origin such as oropharyngeal mucosae, crevicular fluid, blood-derived compounds, and food debris [1, 2]. Typically, the collection and evaluation of secretions from individual salivary glands are used for the detection of gland-specific pathology such as infection and obstruction. However, due to its easy sampling method, with or without stimulations, whole saliva is more frequently studied especially for the evaluation of systemic disorders [3].

Generally, saliva sampling involves a simple and noninvasive collection method that allows easy storage and transport [2]. This painless procedure is particularly useful for people with problems in collecting blood samples such as haemophiliacs, neonates, elderly people, and disabled people among others [4]. In addition, it also increases the compliance of people who require frequent clinical monitoring with multiple sampling over the day or several days, thus increasing the feasibility for monitoring their health progression and treatment outcomes [5]. Unlike blood specimen, saliva sampling does not require specialised instruments or trained personnel with phlebotomy skills, it has minimal or no risk of crosscontamination among patients and offers very low exposure of healthcare personnel to blood-borne pathogens such as HIV and hepatitis [5, 6]. However, it is important to standardise the method of collection in order to obtain significant results.

To date, a wide spectrum of compounds present in saliva has emerged as highly informative and discriminatory. These biomarkers might aid in (i) early detection and diagnosis of diseases; (ii) supporting treatment decision making; and (iii) monitoring disease progression and/or treatment outcomes. These biomarkers have been previously studied by employing conventional collection and laboratory-based assay methods. Although saliva sampling using oral fluid collectors and commercial devices is generally safe and convenient to use and provides sufficient homogeneous sample with low viscosity, it still presents several shortcomings such as (i) the requirement of supervision; (ii) the need to follow the procedures carefully to ensure sample adequacy; and (iii) the relatively time-consuming process (~1-2 min) [7]. On the other hand, salivary analysis using laboratory-based assay methods often requires relatively large volumes of sample and involves multiple steps of sample acquisition, labelling, freezing, transportation, processing in the laboratory (e.g., centrifugation, sorting, aliquoting, and loading into the analyser), analysis, and, finally, results reporting. It is a tedious and lengthy process, in which each step needs to be performed carefully as it is fraught with several potential quality failure points. In such scenarios, saliva sample storage procedures between sampling and analysis also need to be taken into account, as it may affect the relative stability of the salivary components. In terms of financial implications, the analytical instruments utilised are expensive, hence available in centralised laboratories only. There are also costs associated with the testing supplies, sample acquisition, and transport supplies, as well as the labour costs incurred across the total process. The aforementioned drawbacks have led to the demand for rapid and reliable quantification of salivary biomarkers through the use of biosensing technology [8]. The ability to immediately collect and analyse salivary biomarkers on site (point-of-care (POC)) provides countless advantages for clinical applications.

Biosensors are small, self-contained analytical devices used for the detection and measurement of a particular substance (analyte) of interest. A biological sensing element (e.g., enzymes, antibodies, nucleic acids, etc.) is placed in intimate contact with a transducer (e.g., optical, electrochemical, piezoelectric, etc.) transforming the biorecognition event into a more simple and quantifiable signal. Generally, the strength of the output signal is proportional to the concentration of the analyte of interest. Finally, the result is processed using associated electronics and embedded software systems, which provide simple digital feedback displayed using a reader device in a user-friendly manner for interpretation by nonexperts [5]. However, it is noteworthy that the reader device accounts for the most expensive part of the sensor, so it is normally incorporated in sensors that are intended for commercial purposes. For a comprehensive description of the progress in biosensors for medical applications with specific emphasis in noninvasive measurement the following book chapter is recommended [9].

This paper reviews the significance of salivary biomarkers for clinical diagnosis and therapeutic applications. Each biomarker and its associated detection methods present different particularities, drawbacks, and challenges. Hence, the most common salivary biomarkers and their biosensing mechanisms have been described separately focusing on the reported technologies used for both the collection and the screening of these biomarkers.

## 2. Salivary Glucose

As glucose is a small organic molecule, it has the ability to move easily through the blood vessels membranes, diffusing from the blood plasma to the gingival fluid via the gingival sulcus into saliva. There are many controversial findings regarding the correlation between capillary blood glucose (CBG) and salivary glucose (SG) concentrations in the literature. In healthy subjects, there is a lack of relationship between CBG and SG concentrations because insulin from beta cells in the pancreas is released into the bloodstream to normalise the CBG level when the glucose concentration exceeds a certain limit [4, 10]. In diabetics, SG concentration is significantly higher than in healthy subjects [4, 11–17]. However, the association between CBG and SG levels among diabetics is ambiguously established as there are contradicting studies that found both positive correlation [4, 11, 12, 16] and absence of [14, 17] correlation, hence making the use of SG concentration as an index for diabetes mellitus inconclusive.

Gingival crevicular fluid (GCF) is an extracellular fluid secreted from the epithelia of the gingival crevice, a V shaped crevice surrounding all teeth, which is located between the tooth and the gingiva with a clinically normal depth of 0.5 to 2.0 mm [18, 19]. In the last decade researchers have investigated the possibility of using gingival crevicular blood (GCB) as an assessment method to measure glycaemia among diabetics. The results revealed that there is a significant correlation between GCB glucose and CBG concentrations [20–28]. During periodontal inflammation, with or without the complicating factor of diabetes, an ample amount of extravasated blood is produced [29]. Since periodontal disease is prevalent among diabetics, the GCB collected with the probing technique during a general periodontal examination can be used to diagnose diabetes or to monitor CBG level in known diabetics. This technique is inexpensive, safe, and easy to perform without the use of additional tools such as the sharp lancet for finger puncture. Hence, GCB concentration is suitable for noninvasive CBG assessment, especially in periodontal management among diabetics [20–28].

The first glucose biosensor was developed by Clark and Lyons [30]. The biosensor consisted of an electrode that monitored the consumption of oxygen (O_2_) catalysed by glucose oxidase (GOx) enzyme as shown in

(1) Glucose+O2→GOxGluconic  acid +Hydrogen  peroxide    (H2O2)

In order to determine SG concentration Yamaguchi et al. [31] developed a system that combined flow injection analysis and an O_2_ electrode. The O_2_ consumed was detected amperometrically and as the reaction progressed, a decrease in the output current was observed due to the reduction in the concentration of dissolved O_2_ that reached the sensor (see (1)). A constant current proportional to the glucose concentration in the sample solution was generated when the amount of O_2_ consumed on the enzyme membrane was in equilibrium with the amount of O_2_ released from the sensor. The system allowed monitoring the time-course changes of SG level in the range of 0.1 to 10 mg dL^−1^ using 200 *μ*L of sample solution. The system was further improved by replacing the O_2_ electrode with a hydrogen peroxide (H_2_O_2_) electrode in the flow cell [32]. In this glucose sensor, an increase in the concentration of H_2_O_2_ was observed as the reaction (see (1)) progressed, resulting in an increase in the output current. A constant current proportional to the glucose concentration in the sample solution was generated and detected using an amperometric circuit. The use of H_2_O_2_ electrode type glucose sensor was more desirable than O_2_ electrode type glucose sensor because it reduced the influence of dissolved O_2_. The H_2_O_2_-based saliva analysing system required only 50 *μ*L of sample solution and it also enabled the monitoring of time-course changes of SG level in the range of 0.1 to 10 mg dL^−1^.

Another approach reported GOx enzyme immobilised on a ferrocene modified gold (Au) film electrode using glutaraldehyde (GA) for the crosslinking method [33]. Ferrocene derivatives are recognised as good electron shuttles. Hence, they are commonly used to eliminate O_2_ levels dependence and electroactive biological species interference with the sensor signal. The sensor reported working range for glucose measurement from 0 to 2.2 mM with sensitivity of 21.45 nA *μ*mol^−1 ^cm^−2^ and detection limit (DL) of 1 *μ*M within a response time of 5 s. In these studies saliva samples were collected using a conventional sampling method (dental cotton rolls) and subsequently analysed using the previously discussed SG sensors. However, in the following investigations, sampling devices were developed for integration with their respective sensors.

**Figure 1:**
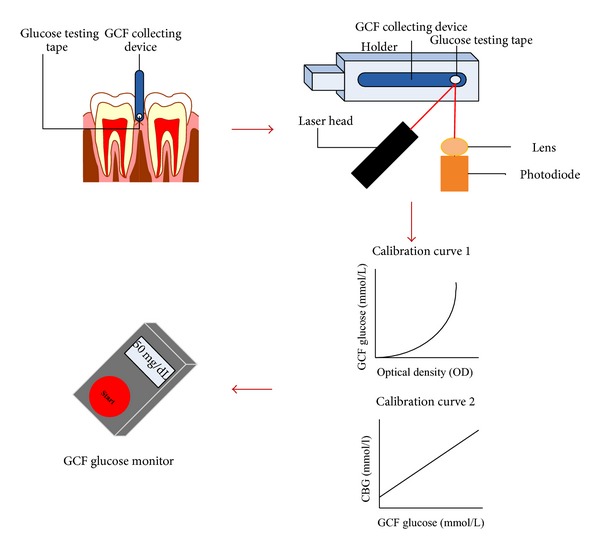
The working principle of the GCF glucose monitor.

Since GCB glucose concentration was also reported suitable for noninvasive CBG assessment [20–28], research was carried out towards the development of diagnostic devices to collect and analyse GCF glucose level [19, 34, 35]. The noninvasive GCF glucose monitoring system comprised a disposable GCF-collecting device, glucose testing tape (GTT), and portable optical analyser [34, 35]. The working principle of the device is illustrated in Figure 1. The GCF collecting device was made of three sheets of laminated polyester film that led the sample solution automatically into the built-in GTT integrated with chromogen (3, 3′, 5, 5′-tetramethylbenzidine (TMBZ)). In the presence of glucose H_2_O_2_ was generated (see (1)) and this in turn oxidised the TMBZ. This reaction is catalysed by peroxidase (POx) enzyme resulting in a colour change on the GTT from white to bluish green:

(2) H2O2+TMBZ →POxColour  change(White⟶Bluish  green)

The colour intensity corresponding to the GCF glucose level in the solution was then measured and the final output was displayed as CBG concentration on the GCF glucose monitor. The device was further modified to enable visual confirmation of the sample collection and to reduce the sample volume size [19]. When GCF sample was collected using the modified GCF collecting device (made of black polyester films), a colour change from white to black was observed, hence confirming the completion of the GCF sampling process. Besides that, the volume of the sample was fixed to be below 200 nL by reducing the tape size. Compared to previous methods, which integrated the GCF collecting device with a GTT saturated with enzyme and chromogen [34, 35], the new GCF collecting device was used to merely collect the GCF sample alone, whilst the analysis was carried out separately by using combined microplate fluorometer and luminometer. The reduced GCF sample collection time (from about 1-2 min to 5–30 s only) and GCF sample evaporation, as well as its increased accuracy, repeatability, sensitivity, and specificity, are additional advantages of this device. Hence, it has the potential to be used as a screening method for noninvasive CBG measurement among diabetics. Furthermore, the application of the reported method can be further extended for the collection and analysis of other biological fluids produced in small quantities for the diagnosis of various other diseases [19].

Overall, since the correlation between CBG and SG concentrations still raises controversy, it would be preferable to develop new biosensing technologies that focus on the measurement of GCF glucose concentration. A possible platform could be one that combines both sampling and analysis using a cellulose fibre-based hand-held device. For instance, a type of dental floss that allows GCF sample collection, followed by immediate sample transcription to the device via capillary action and, finally, obtaining the GCF and/or CBG glucose measurement instantaneously. This simple and noninvasive method could revolutionise the technology in diabetes care.

## 3. Salivary Lactate 

At low oxygen levels cells are forced to metabolise glucose anaerobically leading to the production of lactic acid as a primary by-product. During elevated levels of lactic acid (hyperlactatemia) in blood a significant drop in the blood pH occurs. This physiological condition is known as lactic acidosis. Hence, it is important to monitor capillary blood lactate (CBL) concentration, especially among patients in intensive care units and operating rooms as lactic acidosis can lead to muscle damage that may result in heart attack [36]. It is also essential to measure CBL levels among diabetics due to the close metabolic relationship between glucose and lactate [36, 37]. Besides that, analysis of CBL concentration is of high interest in sports medicine for athletes to tailor their training in order to optimise their performance [38, 39]. For instance, it is important to determine the anaerobic threshold (AT) or maximum lactate steady state (MLSS) from the lactate load curves during exercise, which is the maximum load when lactate production is in equilibrium with lactate elimination. Based on the AT, suitable exercise intensity can be precisely given to the athletes for different types of sports in order to produce optimal results [38].

Lactate can also be detected in saliva due to the passive diffusion of lactate from blood and secretion from salivary glands [40]. The typical lactate concentration in saliva ranges from 0.1 to 2.5 mM [38]. Since SL has a high correlation to CBL concentration, typically a 1 : 4 saliva/blood ratio, SL is suitable for noninvasive CBL analysis [41], especially for critical-care patients, diabetics, and athletes [38, 42].

Lactate biosensors follow the same principle as their glucose counterparts, since they also rely on oxidase enzyme reaction for the biorecognition process. In brief, lactate is oxidised into pyruvate by lactate oxidase (LOx) enzyme consuming O_2_ and producing H_2_O_2_:

(3) $$\begin{array}{c} \text{Lactate}+{\text{O}}_{2}\overset{\text{LOx}}{\to }\text{Pyruvate}+{\text{H}}_{2}{\text{O}}_{2} \end{array}$$

Several biosensors for SL measurements have been reported. One of them is consisted of an electrochemical surface-type sensor that was incorporated with dual platinum (Pt) electrodes (one active lactate probe and the other inactive to lactate) and a common silver/silver chloride (Ag/AgCl) reference electrode developed on Plexiglas [4, 42]. The enzyme membrane was prepared by immobilising LOx enzyme using crosslinking method with GA. After drying, the enzyme membrane was cut into two pieces. One piece was placed on one of the platinum electrodes (active lactate probe) and the other one was immersed in boiling water for 1 min before being placed on the other platinum electrode (inactive to lactate). The use of dual platinum electrodes helped to evaluate and eliminate changes in background current due to pH, temperature, and ionic strength variation, as well as current changes due to potential interferent substances. To improve the permselectivity of the sensor, cellulose acetate and polycarbonate membranes were used as the inner and outer blocking membranes, respectively. The current changes due to the production of H_2_O_2_ (see (3)) proportional to the lactate concentration in the sample solution were measured at 0.65 V versus Ag/AgCl using an electrochemical detector. The reported sensor required the measurement to be conducted in a bulk stirred solution that needed a relatively large volume of sample and thus feasible in laboratory-based analysis only. Hence, there was a need for miniaturised SL sensors that allowed location independent and if possible continuous real-time SL measurements.

**Figure 2:**
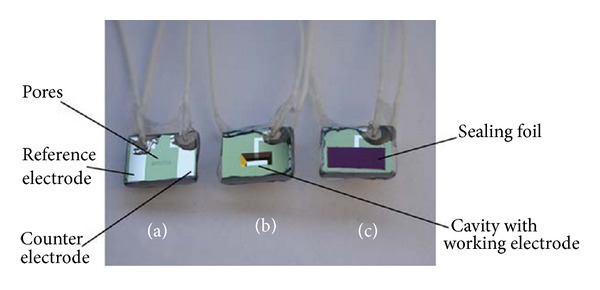
Photograph of the packaged lactate chip. (a) The front side showing the pores, the Ir/IrOx reference, and counter electrode. (b) The back side with cavity and Pt working electrode. (c) The back side covered with sealing foil after filing. Reprinted from [38] with permission from Elsevier.

Schabmueller et al. [38] reported a miniaturised cavity-type sensor chip with three-electrode configuration that was built using silicon microfabrication technology for continuous SL monitoring. The sensor has a cavity with fine pores on its floor, in which LOx enzyme was immobilised in a matrix of agarose gel and sealed by a self-adhering polyester foil (Figures 2(b) and 2(c)). When the sensor was immersed in a sample solution, lactate molecules diffused through the pores in the floor into the cavity, whereby the typical lactate enzymatic reaction (see (3)) occurred at 0.5 V versus iridium/iridium oxide (Ir/IrOx), resulting in an amperometric readout corresponding to the lactate concentration in the sample solution. The sensor was optimised for a linear range of lactate measurement from 0.01 to 5 mM with sensitivity of 78 nA mM^−1^. The sensor was envisioned for real-time* in vivo* monitoring of SL by placing it inside the mouth during exercise and monitoring the results in a portable wireless manner [43].

Another type of sensors developed for SL measurement was based on the principle of electrochemiluminescence (ECL) [36]. The disposable optical sensor employed a LOx based enzymatic recognition system and luminol as the transduction system. In brief, the sensor was prepared by immobilising all the required reagents in a methocel cellulose membrane on a commercial screen-printed electrode (SPE). In the presence of lactate (see (3)), the produced H_2_O_2_ reacted with the electrochemically oxidised luminol, resulting in ECL emission that was measured by a photocounting head. The sensor exhibited dynamic range of lactate detection from 0.1 to 0.5 mM with DL of 5 *μ*M within a short measurement time of 20 s. Since the detection range was not within the physiological range of SL (0.1 to 2.5 mM) [38], the saliva sample required dilution (1 : 4) with the working buffer before the analysis. In addition, the filtration of the saliva sample, a pretreatment (0.4 V for 4 s) to oxidise ascorbic acid, and the application of a blank procedure to correct the error from the presence of uric acid were necessary to reduce the effect of interferent substances.

More recently, a mouthguard sensor based on printable Prussian-blue (PB) transducer and poly-orthophenylenediamine (PPD)/LOx reagent layer was reported for continuous SL monitoring [44]. The extremely low detection potential for H_2_O_2_ (0.042 V versus Ag/AgCl) provided by the PB layer and the permselective behaviour of the PPD layer minimised the effect of possible interferences that are commonly present in saliva matrix. The sensor displayed dynamic range for lactate determination from 0.1 to 1 mM with sensitivity of 0.553 *μ*A mM^−1^ and DL of 50 *μ*M. It also exhibited good stability for continuous SL measurement (2 h duration with repeated measurement every 10 min) due to the PPD layer that protected the sensor surface against coexisting fouling constituents. The device was proposed as a practical wearable sensor that could always be in contact with saliva for continuous noninvasive monitoring of lactate in real time.

Since the material and fabrication process employed in constructing the aforementioned SL sensors were relatively expensive, complicated, and inappropriate for applications in the developing world, our group [45] developed a cotton fabric-based electrochemical device (FED) based on carbon modified with PB (C-PB) electrodes and LOx enzyme for SL measurement. The device was fabricated using a simple template method for patterning the three-electrode configuration and wax patterning technique to create the sample placement/reaction area on the cotton fabric substrate. The FED exhibited a linear working range for lactate detection from 0.1 to 5 mM with sensitivity of 0.3169 *μ*A mM^−1^ and DL of 0.3 mM. Since the fluid flow in the cotton fabric platform occurred via capillary forces, it was envisioned that the use of pipettes could be eliminated by incorporating a hydrophilic cotton thread or an extension of the cotton fabric to enable both sampling and analysis within a single device.

Since continuous assessment of lactate is essential for clinical applications and sports monitoring, two of such wearable sensor concepts [38, 44] based on saliva samples have been reported in the literature. However, critical assessment of all potential toxicity and biocompatibility concerns should be addressed in order to allow it to be adapted as a safe and practical wearable sensor to be placed inside the mouth [44]. In addition, the sensors should also be easily removable and washable to maintain good oral hygiene. In order to make it feasible for long-term continuous SL monitoring, it is also crucial for the sensors to exhibit high operational stability. Finally, the sensors should be integrated with amperometric circuits and electronics for data acquisition, signal processing, and wireless transmission to provide real-time information of the wearer's health status.

## 4. Salivary Phosphate

Salivary phosphate (SP), via saliva secretion and swallowing, provides a source of endogenous phosphate, which at high levels can contribute to hyperphosphatemia. This in turn can result in cardiovascular calcification among chronic renal failure patients, which can lead to high morbidity and mortality, especially among haemodialysis patients. Therefore, it is important to control serum phosphate through the monitoring of SP level [46–48]. In addition, the significant relationship existing between SP and oral health can be used to predict the development of dental caries and formation of dental calculus [49]. SP level is also used as an indicator of ovulation, which aids women to predict their fertile period, especially for the treatment of infertility [50, 51].

The conventional instrumental methods for phosphate measurement include spectrophotometry and chromatography. However, these methods are tedious and time-consuming and need expensive instruments. They also require sample pretreatment and the use of carcinogenic chemicals, such as molybdenum and antimony tartrate, that can affect human health and the environment [52]. Therefore, an amperometric biosensor using pyruvate oxidase (PyOx) enzyme as the biorecognition molecule was developed for rapid SP analysis [53]. The PyOx enzyme was immobilised by a Nafion matrix and covered by a poly(carbamoyl) sulfonate hydrogel as a protective layer on the SPE with two-electrode configuration. The presence of phosphate in the sample solution causes the enzymatic generation of H_2_O_2_ (see (4)), which results in a current difference proportional to the concentration of phosphate in the sample. The results were monitored using a potentiostat at 0.42 V versus Ag/AgCl. The sensor has a short response time and recovery time of 2 s and 2 min, respectively, as well as a short measurement time of 4 min. This provides rapid phosphate analysis compared to the 10 min required for measurement using a commercial phosphate testing kit. The sensor also demonstrated linear working range for phosphate measurement from 7.5 to 625 *μ*M with sensitivity of 523.89 nA mM^−1^ and DL of 3.6 *μ*M. Consider

(4) Phosphate+Pyruvat+O2→PyOxAcetylphosphate   + H2O2   + Carbon dioxide (CO2)

Although SP measurement is important for clinical purposes, there were limited SP biosensors reported in the literature. Due to its serious environmental implications phosphate research has been focused mainly on the monitoring of phosphate concentration in water [52]. Nevertheless, given the promising applications of SP detection, this field of research presents potential for further exploration.

## 5. Salivary Alpha-Amylase

One of the most important enzymes in saliva is salivary alpha-amylase (sAA), which plays an important role in the initiation of carbohydrate digestion in the oral cavity. It also binds specifically with high affinity to several oral streptococci leading to bacterial clearance and nutrition that has important implications for dental plaque and caries formation in the oral cavity [54, 55]. sAA has been identified as an important stress biomarker that increases in response to both physical and psychological stress via interactions with the autonomic nervous system, thus making it useful as a biomarker of autonomic dysregulation [54]. Consequently, it is used to measure the effects of stress-reducing interventions [56, 57]. sAA concentration is also utilised to determine the AT during exercise training as an alternative to SL measurement [58]. In addition, it is used as a quantifiable biomarker for conditions such as pain for objective assessment of pain intensity [59] or for detecting sleepiness [60]. Generally, the physiological range of sAA in normal subjects is around 10 to 250 U mL^−1^ [61].

In the literature, various strategies have been reported to determine sAA activity using biosensing technology. For example, Yamaguchi et al. [62] used a flow-injection type device to continuously supply the substrate, maltopentaose; meanwhile two enzymes *α*-glucosidase (GD) and GOx were incorporated to measure the sAA activity based on (5), (6), and (1). Briefly, AA enzyme in the saliva sample hydrolyses the 1,4-*α*-D-glucosidic linkages in the maltopentaose resulting in maltose and maltotriose (see (5)). This is followed by the reaction of GD enzyme with the prior products to produce five molecules of D-glucose (see (6)). The typical glucose enzymatic reaction (see (1)) then takes place resulting in H_2_O_2_ that was measured using a potentiostat at 0.6 V versus Pt counter electrode. Conisder

(5) $$\begin{array}{c} \text{Maltopentaose}\overset{\text{AA}}{\to }\text{Maltose}+\text{Maltotriose} \end{array}$$

(6) $$\begin{array}{c} \text{Maltose}+\text{Maltotriose}\overset{\text{GD}}{\to }\text{5D-glucose} \end{array}$$

The system enabled the measurement of sAA activity from 0 to 30 U mL^−1^. In addition, a disposable device, which collects saliva in about 90 s using capillary action, was also fabricated to eliminate saliva pretreatment step. The reported system was not designed as a standard device with a working enzyme electrode but as a large analytical system with complicated construction. In the following year, Zajoncová et al. [63] introduced a relatively simple flow-injection method without precolumn and reduced analytical time by adapting mutarotase (MR) enzyme. This utilised a peroxide electrode equipped with three different enzymes, GD, MR, and GOx that were immobilised on a cellophane membrane. In summary, when maltose, which was produced via starch hydrolysis (see (7)), reacts with GD enzyme, it results in two molecules of *α*-D-glucose (see (8)). This is then converted to *β*-D-glucose by MR enzyme (see (9)). Finally, the *β*-D-glucose produced was measured via the glucose enzymatic reaction (see (1)) that produces H_2_O_2_ at 0.65 V versus Ag/AgCl electrode. Consider

(7) $$\begin{array}{c} \text{Starch}\overset{\text{AA}}{\to }\text{Maltose}+\text{Maltotriose}+\text{Dextrin} \end{array}$$

(8) $$\begin{array}{c} \text{Maltose}\overset{\text{GD}}{\to }2\alpha \text{-D-glucose} \end{array}$$

(9) $$\begin{array}{c} \alpha \text{-D-glucose}\overset{\text{MR}}{\to }\beta \text{-D-glucose} \end{array}$$

The biosensor has DL of 2 nkat mL^−1^ and 0.5 nkat mL^−1^ at amylase reaction time of 5 min and 30 min, respectively. It also exhibited a linear range of current response from 0.1 to 3 mM maltose with a response time of 35 s. Without MR, the biosensor was stable for at least two months and retained 70% of its original activity, but the stability was decreased to only 3 weeks with MR. The storage (dry state at 4°C) lifetime of the biosensor was approximately 6 months without MR, but only 3 months with MR. However, the biosensor containing MR provided a smaller relative error (3.5% for pancreatic amylase or 3.9% for sAA) compared to the one without MR (6.7% for pancreatic amylase or 7.1% for sAA).

In order to enable location independent mental stress evaluation, the development of portable sAA activity monitoring devices based on optical detection method was investigated [64–66]. The first prototype of the portable monitor comprised a disposable testing strip, saliva transcription device, and an optical analyser [66]. The disposable testing strip consisted of a collecting strip for saliva sample collection and a reagent strip incorporated with Gal-G2-CNP (2-chloro-4-nitrophenyl-4-O-*β*-D-galactopyranosylmaltoside) chromogen on a testing tape to measure the sAA activity. The described system required saliva sample to be collected manually using a cotton roll, then condensed using a medical syringe, and applied on the collecting strip. When the saliva transcription device was bent, the collecting strip and reagent strip were in contact with each other, hence allowing the sample to be transcribed from the collecting strip to the reagent strip. At the reagent strip, 2-chloro-4-nitrophenyl (CNP) was hydrolysed in the presence of AA, resulting in a yellow coloured product as shown in

(10) GAL-G2-CNP→AAGAL-G2 +CNP (White⟶Yellow)

**Figure 3:**
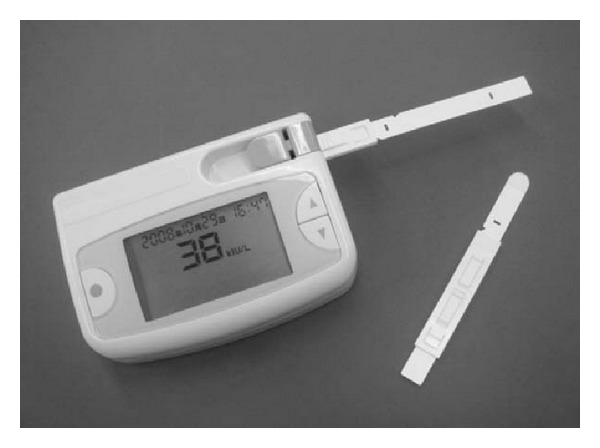
Portable sAA biosensor comprised hand-held reader and a disposable collector strip. Reprinted from [64] with permission from Elsevier.

The enzyme reaction would continue until all the substrate was consumed. The colour intensity corresponding to the changes in sAA activity was then measured by inserting the reagent strip into the optical analyser. The device was able to measure sAA activity from a range of 0 to 200 U mL^−1^ by using only 5 *μ*L of saliva sample within 150 s. In the subsequent work [65], a completely automated sAA activity system was fabricated using a disposable test-strip equipped with built-in collecting and reagent papers as well as an automatic saliva transfer device. The collecting paper was directly inserted into the oral cavity for collection of whole saliva sample and this was immediately placed onto the automatic saliva transfer device. When a lever was operated, the reagent paper attached to the back of a spring of the sleeve was pushed towards the collecting paper, hence resulting in saliva transfer from the collecting paper to the reagent paper. An alarm was triggered when the saliva transfer was complete and the sheet was pulled out of the sleeve, allowing the reflectance to be measured by the optical device. The hand-held monitor took 30 s for saliva sampling and another 30 s for saliva transfer and measurement, hence allowing measurements of sAA activity within a total of 1 min by using only 30 *μ*L of saliva sample. The system demonstrated a linear range of sAA activity between 10 and 140 U mL^−1^. In order to produce a portable, POC biosensor system for rapid measurement of sAA level, the above-discussed sAA optical monitors were technically improved to allow ambulatory care by (i) reducing its overall size; (ii) including temperature-stable test strips for extended storage time at room temperature; (iii) incorporating pseudosubstrates to slow down the enzyme kinetics to provide an extended dynamic range (5 to 450 U mL^−1^) with linear range from 10 to 230 U mL^−1^; (iv) including improvised collector pads to control the sample volume; and (v) integrating refined algorithms for data processing and digital time/date stamps for the collected data (Figure 3). The new prototype provided fast sample acquisition (~10 s), eliminated sample preparation steps, and offered rapid reporting (within 30 s) by using only 25 *μ*L of saliva sample. In addition, the use of disposable plastic collector strips for saliva sample collection was cost-effective and reduced handling disadvantages caused by commercial saliva collection devices [64].

The need for a technology that is small and wearable has also led to the development of a device with a new design. This consisted of a flat-chip microanalytical enzyme sensor that behaved as a microelectromechanical system (MEMS) [67]. In order to miniaturise the flow cell, two enzymatic membranes, containing maltose phosphorylase (MP) and a combination of GOx and POx, were immobilised within the same planar surface. Similar to previous work [62], maltopentaose was used as the substrate and a flow-injection system was adapted as the analytical system. The resulting maltose from the AA enzymatic reaction (see (5)) further reacted with MP enzyme to produce *α*-D-glucose (see (11)). Finally, the *α*-D-glucose undergoes the typical glucose enzymatic reaction (see (1)) with POx as a mediator and the resulting current was measured at 0.6 V versus Pt counterelectrode. The sensor was able to measure sAA activity from 0 to 190 U mL^−1^ by using only 50 *μ*L of saliva sample. Consider

(11) α-Maltose+Phosphate→MPα-D-glucose +β-D-glucose-1-phosphate

Using a different approach, Aluoch et al. [68] introduced an amperometric biosensor based on sAA antibody (Ab) and antigen (Ag) interaction. The biosensor comprised a salivary Ag (or Ab) layer self-assembled onto Au electrode via covalent attachment. The molecular recognition between the immobilised Ag (or Ab) and the sAA proteins was monitored with a monodispersed silver layer on the Au electrode, which acted as the electroactive probe. The biosensor exhibited a linear range for the determination of AA between 3 × 10^−3^ and 1.6 × 10^−2^ ng mL^−1^. The DL for the amylase-Ag immobilised format was 1.57 pg mL^−1^, while for amylase-Ab immobilised format (1/10,000 dilution of amylase Ab) it was 80.9 ng mL^−1^. Another alternative using low cost self-sensing piezoelectric microcantilever biosensor was also reported [69]. The sAA activity was monitored by measuring the amount of deflection of the microcantilever beam in response to the presence of sAA molecules. The corresponding biochemical signal was then converted to voltage by using Wheatstone bridge circuit as the transducer. Although the study showed that the biosensor had capabilities to measure sAA, the device was not tested using real saliva samples.

Another development was a screen-printed disposable sAA biosensor based on Fc as the electron transfer mediator [70]. The working principle of the biosensor relied on the enzymatic reaction of AA on starch and GD on the generated maltose, as in (7) and (8), respectively. Finally, (1) took place assisted by Fc at 0.23 V versus saturated calomel electrode. Both of the enzymes (GD and GOx) were immobilised on the SPE with Nafion film using indirect crosslinking method with GA-bovine serum albumin (BSA). The biosensor exhibited a linear response range for the determination of AA from 60 to 840 U L^−1^ with DL of 17 U L^−1^ and a response time of less than 30 s.

Mahosenaho et al. [71] also used similar enzymes as [63], in which the GD, MR, and GOx enzymes were coimmobilised using crosslinking method with GA-BSA on SPE modified with PB. In this biosensor, several starch substrates (partial hydrolysed starch, amylose, and maltopentaose) were studied, but maltopentaose was chosen due to its high solubility and low molecular weight, which improved the specificity of the enzyme. The reactions that occurred at the biosensor for the measurement of sAA activity are in the sequence of (5), (8), (9), and (1). The biosensor exhibited a wide linear range for the determination of AA from 5 to 250 U mL^−1^ with DL of 5 U mL^−1^. It also demonstrated good operational and storage stability.

In a more recent study, an immunosensor based on the immobilisation of a specific Ab for sAA onto an electropolymerised graphite electrode coated with poly(3-hydroxyphenylacetic acid) was developed [72]. The biomolecular interaction (affinity of the sAA Ab/Ag) in the device was measured using electrochemical impedance spectroscopy (EIS) as the transducer, in which a significant modification in the Nyquist plot was observed upon addition of the complementary target with an increase in the charge transference resistance. The immunosensor presented the potential for the production of a label-free sAA biochip, unlike the aforementioned biosensors that utilised enzymes and chromogen.

It is noteworthy that most of the developed electrochemical transducers for carbohydrate sensing are amperometric biosensors. However, conductometric biosensors have also demonstrated their advantages including the simplicity of the technology to develop reference electrodes, the possibility for miniaturisation, and the utilisation of small amplitude voltage. As a result, a conductometric biosensor array was proposed recently for simultaneous determination of carbohydrates (maltose, lactose, sucrose, and glucose) by Soldatkin et al. [73]. However, conductometric biosensors measurements are susceptible to nonspecific signals from the presence of charged substances. Although this limitation significantly affects the biosensor's accuracy and sensitivity, conductometric biosensors should still be considered for economic reasons.

Compared to other salivary biomarkers, an extensive research adapting different biorecognition molecules and transducers for the development of sAA biosensors has been reported in the literature. This is due to the fact that sAA evidences an excellent capability to quickly and sensitively respond to psychological stress changes, thus enabling it to be used as an important index of the sympathetic nervous system. The above-discussed biosensors exhibited rapid saliva sampling-reporting cycle (~1 min), hence providing a temporal resolution that is useful for time-series psychophysiological measurements in naturalistic settings. In addition, the low saliva sample volume required for the sAA measurement also allowed the system to be used among xerostomic individuals. However, it would be highly advantageous if future research were directed towards the development of miniaturised, wearable sAA biosensors to enable real-time psychological monitoring. The analytical parameters of some of the catalytic salivary biosensors reported in the literature are summarised in Table 1.

## 6. Salivary Hormones

Several hormones that are measurable in plasma can also be detected in saliva including steroids, nonsteroids, peptides, and protein hormones.

Cortisol is a nonpolar steroid hormone that can diffuse through the lipid membranes of salivary glands, which allows a good correlation between serum free cortisol and salivary cortisol (SC) levels [74]. The measurement of SC is useful as a key indicator of stress similar to sAA [75], for physiological studies [76, 77] and screening of Cushing's syndrome [78, 79]. Paradoxically, there is a lack of association between values of serum and salivary cortisone levels even though it is a neutral steroid that readily diffuses into saliva. Salivary cortisone concentration is higher than in serum as cortisol is converted to cortisone by 11 *β*-hydroxysteroid dehydrogenase enzyme present in salivary glands, but it does not have any clinical significance [80, 81]. The same enzyme also affects other corticosteroids such as prednisone and prednisolone, causing absence of correlation between serum and salivary levels [82].

Testosterone is an anabolic androgenic steroid that behaves as the principal male sex hormone. It is also present in women enabling the production of female hormone, oestrogen [83]. Similar to other steroid hormones, salivary testosterone (ST) is in free (bioavailable) form as it is neither bound to proteins nor conjugated, allowing it to be measured easily without dialysis process before assaying [84]. The significant correlation between serum testosterone and ST levels supports it as an attractive noninvasive biomarker for assessing serum testosterone concentration [84–88]. It is important to measure ST levels for behavioural research [89], sports endocrinology [90], and diagnosis of medical conditions such as late-onset hypogonadism and testosterone deficiency syndrome [84, 91] and in determining the androgenic function of prostatic carcinoma patients after medical or surgical orchiectomy [92]. Szydlarska et al. [93] also showed that the ratio of salivary androstenedione/salivary testosterone is a good indicator of hyperandrogenism, a primary symptom of polycystic ovary syndrome.

Female sex steroid hormones such as salivary progesterone (SPg) and estradiol concentrations are also measurable in saliva. These are useful for determining menstrual cycle profiles for fertility and pregnancy monitoring among women [94, 95]. The measurement of progesterone concentration is also useful in the diagnosis of certain medical conditions such as luteal phase defects, impending abortion, and ectopic pregnancy that can lead to sterility. In addition, the effect of drugs such as oral contraceptives, superovulatory drugs, oestrogen replacement therapy medication, and gonadotropin-releasing hormone analogues can be closely monitored from progesterone levels [94].

Nonsteroid hormones such as catecholamine can also be identified in saliva ranging from 250 to 800 pg mL^−1^. However, it does not correlate well with serum due to uncertain origin, making it unsuitable as an index for general sympathetic tone [96, 97]. However, dihydroxyphenylglycol, a metabolite of catecholamine, shows a good correlation with plasma level [98]. In addition, salivary melatonin also shows a highly significant and reproducible correlation with plasma melatonin concentration, hence making it a reliable alternative to venipuncture for pineal physiology studies among newborn infants [99].

Due to the large dimensions of protein plasma hormones, it is difficult for them to pass through the salivary glands by passive diffusion. Thus it is challenging to detect these hormones in saliva [1, 2]. However, these hormones are detectable if they are directly produced by salivary glands. An example is leptin, a protein hormone that is produced, stored, and secreted by salivary glands and expressed in the oral mucosa [100].

Several detection techniques have been evaluated to quantify SC concentration, such as chromatography [101, 102], immunoassays [103–105], and optical [106] and electrochemical immunosensors [107]. Since chromatographic and immunoassay techniques are time consuming and expensive and involve laboratory screening methods, they are not attractive for POC diagnostics. Typically, cortisol biosensors are immunosensors, since they use the specificity of Ag-Ab interactions. Nevertheless, in some cases the use of enzymes is still required to provide a measurable analytical signal. For example, an immunoelectrochemical sensor based on alkaline phosphatase (AP) enzyme was developed to measure SC concentration [108]. In this sensor, cortisol captured Abs were immobilised on functionalised microfabricated Au electrodes that were encased within a microfluidic chamber. When the AP enzyme, which was attached to the cortisol Ag via the detector Abs, reacted with p-nitrophenyl phosphate solution, p-nitrophenol was generated and detected as an oxidative peak between 0.9 and 1.1 V versus Ag pseudoreference electrode. The magnitude of the anodic peak in the cyclic voltammetry varied linearly with cortisol concentration and hence this was used to quantify cortisol in real saliva samples. The sensor accurately measured cortisol in the collected saliva samples to a concentration of 0.76 nM (0.21 ng mL^−1^).

In another study, a surface plasmon resonance (SPR) biosensor system, in which cortisol was detected by competitive assay combined with an in-line filtering flow cell, provided results in less than 10 min, making it suitable for analysis in the field and in emergency rooms [109]. Although the system exhibited a detection range between 1.5 and 10 ng mL^−1^ (5.4 and 36 nM) with DL of 1.0 ng mL^−1^ (3.6 nM) for SC measurement, it could be further extended for high-end detection range by increasing the concentration of Ab or diluting the sample solution. Mitchell et al. [74] also developed a microfluidic SPR biosensor system that utilised competitive assay for SC detection. The biosensor employed cortisol-linker conjugate covalently immobilised on the sensing surface as a coating agent. In this system, the sample solution was mixed with the primary Ab and injected into the flow-through biosensor, where it competed with the surface immobilised cortisol for binding to the Ab. To further enhance the biosensor signal, the surface-bound primary Ab was attached to a secondary Ab by increasing bound mass on the surface. In buffer, the system presented DL of 13 pg mL^−1^ with sensitivity of 72 RU mL ng^−1^. The immunoassay was optimised to cover the physiological range of SC between 91 and 934 pg mL^−1^, but higher concentration samples can be diluted if necessary. The immunoassay exhibited DL of 49 pg mL^−1^ and sensitivity of 162 RU mL ng^−1^ in saliva samples. Since only 50 *μ*L of saliva per sample replicate was required, the SC measurement for each sample could be completed in 10 min.

While the described biosensors reported excellent sensitivity, DL, and response time, the need for user-friendly devices has led to the investigation of disposable devices. An immunochromatographic test strip based biosensor, composed of a disposable test strip and monitor, was developed for this purpose [110]. In this biosensor, a GOx-cortisol conjugate was synthesised and used for the specific Ag-Ab reactions. In brief, when a sample solution was introduced on the sample pad, the solution was filtered and the GOx-cortisol conjugate in the conjugate pad was dissolved. At the test-line, the cortisol Ags in the solution were immobilised with cortisol Ab forming an Ag-Ab reaction. Thus, the concentration of the trapped GOx-cortisol conjugate was inversely proportional to the cortisol concentration in the solution. When GOx enzyme assay was applied on the strip, a red coloured band appeared and its intensity was measured using an optical reader. The biosensor allowed cortisol measurement in the range of 1 to 10 ng mL^−1^ within a total measurement time of 25 min. The system demonstrated its potential utility for a SC POC measurement system. Using a similar working concept (competitive reaction between cortisol in the sample solution and a GOx-labeled cortisol conjugate), Yamaguchi et al. [111] reported a cortisol immunosensor based on electrochemical detection method. The measured current was inversely related to the cortisol concentration in the sample solution. The immunosensor allowed SC measurement in the range between 0.1 and 10 ng mL^−1^ with measurement time of 35 min.

Other electroanalytical techniques have been used for the development of immunosensors. Arya et al. [112] reported an impedimetric cortisol immunosensor using dithiobis(succinimidylproprionate) (DTSP) self-assembled monolayer functionalised Au microelectrode array. The immunosensor exhibited linear behaviour for cortisol concentration in the range of 1 pM to 1 *μ*M with DL of 1 pM. The remarkable electrochemical properties of carbon nanotubes (CNT) have led to the use of these in the field of biosensors, such as the immunosensor based on single-walled carbon nanotube (SWCNT) chemiresistive transducer for SC measurement [113]. The CNT were functionalised with a cortisol analog (cortisol-3-CMO-NHS ester) that was ligated with a monoclonal anti-cortisol Ab. The presence of cortisol displaced the anti-cortisol Ab, hence resulting in a corresponding decrease in the resistance/conductance of the CNT-biomolecule hybrid. The immunosensor exhibited an inverse relationship to cortisol concentration over a range of 1 pg mL^−1^ to 1000 ng mL^−1^ that was linear from 1 pg mL^−1^ to 10 ng mL^−1^ with sensitivity in the linear range of 13.97 ng mL^−1^ and DL of 1 pg mL^−1^. It also demonstrated excellent binding selectivity for cortisol, even in the presence of structurally similar steroids such as 21-hydroprogesterone.

Besides exploring properties of the immobilisation matrix, studies have also been focused on improving the sensitivity and stability of immunosensors. More recently, semiconductor inorganic material has been applied as a successful immobilisation matrix [114, 115]. For instance, one-dimensional zinc oxide (ZnO) nanorods (ZnO-NRS) and two-dimensional ZnO nanoflakes (ZnO-NFs) surfaces were modified to immobilise anti-cortisol Ab in order to develop an electrochemical cortisol immunosensor [115]. The sensitivity of the ZnO-NRS and ZnO-NFs electrodes, estimated using electrochemical impedance spectroscopy method, was 3.078 kΩ M^−1^ and 540 Ω M^−1^, respectively. The DL for both the electrodes was obtained at 1 pM.

As in the case of cortisol, testosterone biosensors are immunosensors. Mitchell and Lowe [87] reported a SPR biosensor to measure testosterone in a wide range of matrices. In this study, an oligoethylene glycol linker conjugate of testosterone was synthesised and applied as a coating agent on the sensing surface using covalent bond immobilisation method. The sensing surface was highly stable, hence allowing a high degree of reusability. The biosensor was further improved using both secondary Ab and gold nanoparticle signal enhancement method, which increased the assay's signal sensitivity 12.5-fold compared with primary Ab alone. The enhanced assay demonstrated a linear response range of testosterone detection from 25 to 250 pg mL^−1^ with DL of 3.7 pg mL^−1^ for standards in running buffer and 15.4 pg mL^−1^ in stripped human saliva matrix. The device was capable of measuring ST in male saliva across the broad physiologically relevant range (29–290 pg mL^−1^) in less than 13 min, making it suitable as a noninvasive near real-time ST biosensor.

As concluded from all the reviewed detection techniques in this section, electrochemical immunosensors provided low DL, high sensitivity, and rapid analysis, compared to optical sensors. However, one important issue that has not been investigated is the impact of environmental factors on the antibody stability. Nevertheless, the miniaturisation and automation of electrochemical immunosensors to measure SC level present great potential for POC diagnostics technology.

## 7. Salivary Antibodies

Saliva is also used for the detection of Abs that fight a number of viral, fungal, and parasitic agents, specific bacteria, and allergy reactions. There are two major Ab classes available in saliva, namely, immunoglobulin A (IgA) and immunoglobulin G (IgG). Generally, IgA is synthesised by plasma cells in salivary glands and then exported by an epithelial receptor-mediated mechanism. Meanwhile, IgG is mainly derived from serum via passive diffusion and a minor fraction is originated from glandular, gingival, or tonsillar plasma cells [116].

The bacterium* Helicobacter pylori* is the major cause of gastrointestinal disorders such as chronic active gastritis and duodenal and gastric ulceration [117]. It is also related to the development of gastric carcinoma [118] and mucosal-associated lymphoid tumours [119]. Several studies have confirmed that the concentration of salivary IgG antibodies that combat* H. pylori* can offer a noninvasive screening method of its infection. However, it has several limitations compared to conventional gastric histological and serological studies, thus making it useful only in certain patient subpopulations or in specific clinical contexts [120–122]. Leptospirosis, which is caused by the bacterium* Leptospira*, is another bacterial infection that can be diagnosed from the detection of specific immunoglobulin M (IgM) class antibodies in saliva [123].

Saliva is also widely used as a diagnostic tool for human immunodeficiency virus (HIV) and hepatitis C virus infection as it outweighs blood testing for Ab screening, particularly for home testing [124–130]. Since HIV infection reduces oral defence mechanism and may affect mucosal integrity, HIV patients are highly susceptible to* Candida albicans* fungal infection that causes oral candidiasis disease. The pattern of two subclasses of salivary IgA that includes IgA1 and IgA2 changes during* C. albicans* fungal infection, making it a potential biomarker to monitor the progression of HIV and acquired immunodeficiency syndrome (AIDS) infection [131, 132].

In addition, salivary antibodies concentration also presents diagnostic value for intestinal infection due to parasitic agents such as* Entamoeba histolytica* [133, 134],* Trichuris trichiura*,* Ascaris lumbricoides*, and hookworms [135]. Another prominent infectious parasitic organism is* Toxoplasma gondii* that causes toxoplasmosis disease. The disease is normally asymptomatic in healthy individuals but causes abortion among pregnant patients or mortality in immunosuppressed individuals such as AIDS patients. Stroehle et al. [136] showed that specific anti-*T. gondii* salivary IgG concentration can be used for the diagnosis of toxoplasmosis disease. Besides that, IgG antibodies to Ags of* Taenia solium* metacestodes infection can also be detected in saliva samples of intracerebral cysticercosis patients, thus making it useful for the diagnosis of neurocysticercosis disease [137].

The broad diagnosis range that salivary antibodies can provide has meant that they are seen as potentially useful in clinical settings. As such there has been an increase in the number of studies on biosensor technologies and their development. A piezoelectric quartz crystal immunosensor comprised of a quartz piezoelectric crystal, an oscillator, and a frequency counter was developed to monitor antibodies in diluted human saliva sample [138]. Briefly, the antibodies against human immunoglobulin A (anti-IgA) were immobilised using two different techniques, adsorption and covalent bond method onto the Au electrode surface of the crystal. The crystal was then oscillated at a basic frequency of 9 MHz by an oscillator. Meanwhile, the oscillation frequency, corresponding to the immunoreactions between the immobilised anti-IgA and IgA in the sample solution, was measured using the frequency counter and translated to a computer. The results revealed that the covalent bond immobilisation method has better repeatability compared to the adsorption method. Subsequently, the immunoreactions between the immobilised anti-IgA using the covalent bond method and diluted human saliva IgA were monitored in real time providing a maximum frequency change at about 1000 s. The immunosensor showed a linear relationship between the maximum frequency change of human saliva and the concentration of standard human IgA. Finally, the amount of Abs in human saliva sample measured using ELISA technique was compared to the results obtained for a 100 times diluted solution of the same human saliva sample using the immunosensor. The results were in good agreement, thus confirming the accuracy of the method.

In another study, Au SPEs have been used to construct polytyramine- (Ptyr-) based immunosensors to target* Streptococcus pyogenes* bacteria [139]. The biotinyl tagged whole antibodies against* S. pyogenes* were conjugated to Ptyr amine group via biotin-NeutrAvidin coupling. The sensor showed linear response of 100 to 105 cells per 10 *μ*L in cumulative incubation and 100 to 104 cells per 10 *μ*L in single-shot incubation, which covered the pathogenic load of* S. pyogenes* infection that is around 106 cells mL^−1^. The sensor was also used to detect* S. pyogenes* infection in saliva, in which it exhibited low crossreactivity and high selectivity, thus demonstrating its capability to be used in complex biological samples such as saliva.

Electrochemical biosensors have also been proven to be a promising platform for the detection of target Abs with high sensitivity and selectivity [140, 141]. Recently, electrochemical peptide-based (E-PB) sensors were also fabricated for anti-HIV antibodies detection [140]. Several surface modification strategies have been used to fabricate E-PB sensors to date. McQuistan et al. [142] reported E-PB HIV sensor fabrication using thiolated oligonucleotides as passivating diluents and its applicability was examined in synthetic human saliva. Although the researchers fabricated several sensitive and selective electrochemical biosensors to target salivary Abs detection, there is still a need for improvement. In this case, electrochemical immunosensors construction should be optimised to achieve higher sensitivity.

## 8. Salivary Cancer Biomarkers and Biosensors

Salivary biomarkers have been identified that can potentially provide useful information for clinical diagnosis and prognosis of a variety of cancers (oral, pancreatic, lung, breast, and liver).

The sixth most common cancer worldwide is head and neck cancer with 40% of these cases being cancer of the oral cavity. Due to the direct contact between saliva and oral cancer lesions, various salivary proteomes have been reported in the literature as potential biomarkers for oral cancer detection such as interleukin-8 (IL-8) [143–145], tumour necrosis factor-alpha, and salivary transferrin [146]. On the other hand, salivary soluble CD44 Ag can be used as a biomarker for head and neck squamous cell carcinoma [147], while the serum circulatory tumour markers, Cyfra 21-1 [148, 149], tissue polypeptide Ag, cancer Ag 125 [148], and salivary zinc finger protein 510 peptide [150], were identified as oral squamous cell carcinoma-related salivary biomarkers.

Pancreatic cancer is the fourth leading cause of cancer-related mortality and second most frequent gastrointestinal malignancy. Zhang et al. [151] carried out a prospective sample collection and retrospective blinded validation to evaluate the performance and translational utilities of salivary transciptomic biomarkers for the noninvasive detection of resectable pancreatic cancer. Results showed that a combination of four messenger RNA biomarkers, namely, KRAS, MBD3L2, ACRV1, and DPM1, in saliva supernatant can distinguish pancreatic cancer patients from healthy subjects. In another study, various lung cancer biomarkers present in saliva were identified to differentiate lung cancer patients from healthy subjects [152, 153].

Another example of the use of salivary biomarkers for the diagnosis of systemic cancer can be seen in breast cancer research. A salivary protein, product of the oncogene c-erbB-2 and cancer Ag 15-3 (CA15-3), was reported to be useful for the early detection and measurement of breast cancer patients' response towards chemotherapy and/or surgical treatment of the disease [154–156]. In another study, it was shown that salivary protein factors such as vascular endothelial growth factor, epidermal growth factor, and carcinoembryonic Ag were elevated in breast cancer patients compared to healthy subjects, hence making it a possible biomarker for breast cancer detection [157].

Since saliva biomarkers have shown to be able to diagnose cancer, different biosensor technologies have been investigated to determine those biomarkers. A surface immobilised optical protein sensor was developed to detect IL-8 protein, an oral cancer salivary biomarker [144]. The sensor has similar DL (1.1 pM) to ELISA assay, despite eliminating the common enzymatic amplification employed in commercial IL-8 ELISA assays. In addition, ELISA and the sensor showed similar diagnostic performances when measuring IL-8 in clinical saliva samples. Nevertheless, the sensor demonstrated better capability for detecting true positives in cancer patients, whilst ELISA showed itself to be slightly better at differentiating true negatives. By using confocal optics to reduce the optical noise, the DL of the sensor was further extended to 4.0 fM, which is the lowest reported sensitivity for IL-8 detection. In addition, an excellent linearity of IL-8 concentrations from 6 fM to 12 pM was observed. The high sensitivity of the confocal optical protein sensor makes it suitable to be applied for a variety of biomarkers, especially those present in very low concentrations.

In another study, a SPR biosensor based on thin film Au/zinc oxide (Au/ZnO) was developed using a radiofrequency sputtering system (13.56 MHz) for the detection of CA15-3, a breast cancer biomarker in human saliva [158]. The performance of the thin film SPR system was compared with a Biacore SPR system for the detection of saliva CA15-3. The Biacore SPR system allowed the detection of higher concentrations of saliva CA15-3, but the sensitivity dropped at lower concentrations (<75 U mL^−1^). On the other hand, the thin film SPR system showed a linear response to saliva CA15-3 from 2.5 to 20.0 U mL^−1^, thus covering the relevant saliva CA15-3 concentration range for both healthy individuals and malignant breast cancer patients. Hence, it was deemed suitable for the clinical diagnosis of breast cancer progression.

In a more recent study, a label free fluorescent biosensor was developed based on nuclease assisted target, and recycling thioflavin T- (ThT-) induced quadruplex formation for amplified short DNA species of c-erbB-2 oncogene (T1) [159]. Since ThT signal transducers amplify the signal from short DNA, the sensor was designed for early stage breast cancer detection with high sensitivity, specificity, speed, and low cost compared to other reported methods. As a result, T1 was detected within a linear range of 100 fM to 1 pM with DL of 20 fM.

Due to low levels of cancer biomarker antigens in saliva, there is a compelling need to develop a more accurate, highly sensitive, and specific biosensor. Overall, the use of immunosensors for the determination of salivary hormones, antibodies, and cancer biomarkers can provide relatively quick and easy monitoring for patients that require multiple diagnoses and continuous monitoring. Although more work is necessary to improve the incubation time required and the sensitivity range, these biosensors have the potential to be used as home care devices providing the patient with a preliminary diagnosis that could then be corroborated in clinics and hospitals.

The development of highly sensitive and selective biosensors for the detection of salivary biomarkers has been emphasised for disease diagnosis in effective and noninvasive methods. However, detection of a single biomarker is not sufficient for accurate medical diagnosis. Therefore, efforts have been made to develop assays or biosensors for multiplexed detection of different types of salivary biomarkers. The first multiplexed electrochemical sensor for the determination of salivary biomarkers for oral cancer detection was reported by Wei et al. [160]. Their results proved that the multiplex detection of both IL-8 mRNA and protein helps to provide a more accurate diagnosis for oral cancer detection. Nevertheless, complications have been reported when using multiplexed sensors to detect various salivary biomarkers simultaneously due to low levels of biomarkers in saliva. The integration of microfluidic systems has been investigated to overcome the difficulties of multiplex ELISA methods. For example, a microfluidic biosensor for the determination of three important serum and saliva cancer biomarkers, namely, carcinoembryonic antigen (CEA), cancer antigen 125 (CA125), and Her2/Neu (C-erB-2), was developed using quantum dots (QDs) [161]. The semiconductor QD nanoparticles presented strong intensity and long-term photostability. Hence, they can be used to overcome the sometime indistinguishable target specific signals from low-level salivary biomarkers. Besides that, a study carried out by Jokerst et al. verified that the integration of semiconductor nanoparticle QDs and a multianalytical nanobiochip reduced the DLs by half compared to ELISA. The capability of the system for multiplexing was evaluated via the spatial beads arrangement on the chip. The specific and nonspecific signals were expressed from three different specified beads with Her-2/Neu, CEA, and CA125 antibodies. The nonspecific signal was reported to be less than 5% of the specific signal for all three different beads. Thus, the results demonstrated that the nanobiochip sensor served as a specific and multiplex method for the determination of multiple salivary biomarkers from saliva and serum with sensitive signals and low DL within a short time [161]. Biosensors for multiplexed detection of different types of salivary biomarkers are still in early stage but present the potential to significantly change cancer diagnosis and prevention. The early diagnosis of cancer could greatly reduce the mortality rates of the disease. This would definitely be a significant breakthrough for the fight against cancer, since a salivary biosensor could be easy to use and economical enough to be a home care device or even a wearable technology.

Some of the immunosensors reported in the literature to determine biomarkers in saliva are summarised in Table 2.

## 9. Conclusion

The significance of salivary biomarkers for clinical diagnosis and therapeutic applications has been reported with additional focus on technologies and biosensing platforms for screening these biomarkers.

Most of the biosensors reviewed have reported excellent sensitivity, detection limit, and response time. Moreover, efforts have been made to miniaturise devices integrated with sample-handling. This allows portability, which in turn provides rapid point-of-care diagnosis and reduces costs (travel cost to the hospital and hospital stay), a highly beneficial characteristic especially for applications in developing countries. The devices could also be conveniently used as home care devices, providing the patient with a preliminary diagnosis that could then be corroborated in healthcare settings. However, these miniaturised devices have currently been limited to the sampling and detection of a small group of salivary biomarkers, mainly SG and sAA. This has created a need to produce devices for measuring a wider range of salivary biomarkers. One proposed recommendation is the development of biosensing devices that combine both sampling and analysis within a hand-held device in a user-friendly manner, such as dental floss and toothbrush. This process would not require supervision of a trained healthcare worker, thus allowing it to be easily performed and location-independent.

In some cases, there is a demand for continuous assessment of biomarkers to monitor the patient's health condition and progression after treatment. This has led to increasing interest in wearable sensor technologies that can be placed inside the patient's mouth. However, several factors need to be taken into consideration in the design and implementation of these sensors: (i) potential toxicity and biocompatibility; (ii) easy removability and washability for hygiene purposes; and (iii) high operational stability. Additionally, the sensors should be integrated with circuits and electronics for data acquisition, signal processing, and wireless transmission to provide real-time information of the wearer's health status.

Based on the reported devices, it can be observed that various modifications to the sensors have been investigated in order to provide very low detection limit and highly sensitive results, especially in the case of immunoassays for detection of salivary hormones, antibodies, and cancer biomarkers. Therefore, it is envisioned that the rapidly evolving fields of proteomics, bioengineering, and analytical chemistry will provide an impact on the development of a well-equipped and reliable sensing platform for saliva-based diagnosis in the near future. Meanwhile, the significance of biosensors for multiplexed salivary biomarkers detection in cancer research, which can provide greater accuracy compared to a single biomarker approach, will further fuel the research in this area.

Although biosensors have long been investigated as diagnosis systems, only in the last decade has this research focused more intensely on devices that are wearable, easy to use, and minimally invasive for applications as point-of-care technology. Nanotechnology and materials science have revolutionised the field of clinical devices. Biosensors can now be easily miniaturised; they are more stable and robust and present higher degree of biocompatibility, allowing contact with the sample* in situ*. Since saliva presents good correlation with various plasma biomarkers and it is relatively easy to sample, an increasing number of saliva biosensors investigations can be expected in the following years.

## Figures and Tables

**Table 1:** Summary of the analytical parameters for catalytic salivary sensors reported in the literature.

Biomarker	Biorecognition	Transducer	Sample volume (*μ*L)	Working range	Sensitivity	Detection limit (DL)	Reference
Glucose	GOx	Electrochemical	200	0.1–10 mg dL^−1^	—	—	[31]
	GOx	Electrochemical	50	0.1–10 mg dL^−1^	—	—	[32]
	GOx/Fc∗	Electrochemical	—	0–2.2 mM	21.45 nA *μ*mol^−1^ cm^−2^	1 *μ*M	[33]
	GOx/POx/TMBZ∗	Optical	0.2	0–10 mg dL^−1^	—	—	[34, 35]
	GOx/POx/TMBZ∗	Optical	0.2	—	—	—	[19]

Lactate	LOx	Electrochemical	1000	—	42 nA mM^−1^	—	[4, 42]
	LOx	Electrochemical	—	0.01–5 mM	78 nA mM^−1^	—	[38]
	LOx/luminol∗	Electrochemiluminescent	50	0.1–0.5 mM	—	5 *μ*M	[36]
	LOx/PB∗	Electrochemical	—	0.1–1.0 mM	0.553 *μ*A mM^−1^	50 *μ*M	[44]

	LOx/PB∗	Electrochemical	4	0.1–5 mM	0.3169 *μ*A mM^−1^	300 *μ*M	[45]
Phosphate	PyOx	Electrochemical	50	7.5–625 *μ*M	523.89 nA mM^−1^	3.6 *μ*M	[53]

Alpha-amylase	GD/GOx	Electrochemical	100	0–30 U mL^−1^	—	—	[62]
	GD/MR/GOx	Electrochemical	35	0.1–3 mM	—	50/12 nM (5/30 min)	[63]
	Gal-G2-CNP	Optical	5	0–200 U mL^−1^	—	—	[66]
	Gal-G2-CNP	Optical	30	10–140 U mL^−1^	—	—	[65]
	Gal-G2-CNP	Optical	25	10–230 U mL^−1^	—	—	[64]
	MP/GOx/POx	Electrochemical	50	0–190 U mL^−1^	—	—	[67]
	Amylase Ag-Ab∗	Immunoelectrochemical	—	3 × 10^−3^–1.6 × 10^−2^ ng mL^−1^	—	1.57 pg mL^−1^	[68]
	GD/GOx/Fc∗	Electrochemical	—	60–840 U mL^−1^	—	17 U mL^−1^	[70]
	GD/MR/GOx/PB∗	Electrochemical	—	5–250 U mL^−1^	14 nA U^−1^	5 U mL^−1^	[71]

*The biorecognition molecule is listed together with the chromogen that provides the change of colour for optical determinations with the redox mediators that facilitate electrons transfer for electrochemical determinations.

**Table 2:** Summary of the reported SL immunosensors analytical parameters.

Biomarker	Biorecognition	Transducer	Sample volume (μL)	Working range	Sensitivity	Detection limit (DL)	Reference
Cortisol	Cortisol Ag-Ab/AP	Immunoelectrochemical	30	—	—	0.21 ng mL^−1^	[108]
	Cortisol conjugate-Ab	Optical	—	1.5–10 ng mL^−1^	—	1.0 ng mL^−1^	[109]
	Cortisol-linker conjugate-Ab	Optical	50	91–934 pg mL^−1^	162 RU mL ng^−1^	49 pg mL^−1^	[74]
	GOx-cortisol conjugate/Ag-Ab	Optical	100	1–10 ng mL^−1^	—	—	[110]
	GOx-cortisol conjugate/Ag-Ab	Immunoelectrochemical	40	0.1–10 ng mL^−1^	—	—	[111]
	Cortisol Ag-Ab	Immunoelectrochemical	—	1 pM^−1^ μM	1.165 kΩ M^−1^	1 pM	[112]
	Cortisol analog-anti-cortisol Ab	Field effect transistors/chemiresistors	—	0.001–10 ng mL^−1^	13.97 ng mL^−1^	1 pg mL^−1^	[113]
	Cortisol conjugate-BSA/Anti-cortisol Ab	Immunoelectrochemical	10	100 pM to 100 nM	540 ΩM^−1^	1 pM	[115]

Testosterone	Testosterone-linker conjugate-Ag-Ab	Optical	—	25–250 pg mL^−1^	45 RU mL ng^−1^	15.4 pg mL^−1^	[87]

Immunoglobulin A (IgA)	IgA-anti IgA	Piezoelectric	10 000	—	—	—	[138]
	SAG1 antigen	—	—	—	—	—	[162]
	IgG HIV target	Electrochemical	—	—	—	1 nM	[142]

*Streptococcus pyogenes *(*S. pyogenes*) bacteria	Anti-*S.pyogenes* Ab-Ptyr-biotin-NeutrAvidin	Immunoelectrochemical	—	100 to 10^5^ cells per 10 μL	—	—	[139]

Interleukin-8 (IL-8)	IL-8 capture probe-IL-8 target/Fluorescent	Optical	50	6 fM–12 pM	—	4.0 fM	[144]
	IL-8 capture probe-IL-8 target/Fluorescent	Optical	20	—	—	0.8 pM	[159]

Cancer antigens 15-3 (CA 15-3)	Anti-CA 15-3 Ab	Optical	—	2.5–20.0 U mL^−1^	—	—	[158]
	Anti-CA 153 Ab	Optical	—	0.001–10 U mL^−1^	0.001–0.01 U/mL	0.001 U/mL	[163]
	IL-8 mRNA and protein probe	Electrochemical	—	5 fM–50 pM, 10–12500 pg/mL	3.9 fM 7.4 pg/mL	—	[160]
